# Clinical and patient-centered implementation outcomes of mHealth interventions for type 2 diabetes in low-and-middle income countries: a systematic review

**DOI:** 10.1186/s12966-021-01238-0

**Published:** 2022-01-06

**Authors:** Moses Mokaya, Florence Kyallo, Roman Vangoitsenhoven, Christophe Matthys

**Affiliations:** 1 Department of Human Nutrition Sciences, Jomo Kenyatta University of Agriculture and Technology, Nairobi, Kenya; 2 Clinical and Experimental Endocrinology, Department of Chronic Diseases and Metabolism, KU Leuven, Leuven, Belgium; 3 Department of Endocrinology, University Hospitals Leuven, Leuven, Belgium

**Keywords:** clinical outcomes, mHealth, patient-centered implementation outcomes, type 2 diabetes mellitus

## Abstract

**Background:**

The prevalence of Type 2 Diabetes is rising in Low- and Middle-Income Countries (LMICs), affecting all age categories and resulting in huge socioeconomic implications. Mobile health (mHealth) is a potential high-impact approach to improve clinical and patient-centered outcomes despite the barriers of cost, language, literacy, and internet connectivity. Therefore, it is valuable to examine the clinical and implementation outcomes of mHealth interventions for Type 2 Diabetes in LMICs.

**Methods:**

The Preferred Reporting Items for Systematic review and Meta-Analysis (PRISMA) guidelines were applied in framing and reporting the review criteria. A systematic search of Cochrane Library, Web of Science, PubMed, Scopus, and Ovid databases was performed through a combination of search terms. Randomized Controlled Trials (RCTs) and cohort studies published in English between January 2010 and August 2021 were included. Risk of bias for missing results in the included studies was assessed using the Cochrane risk-of-bias tool for randomized trials (RoB 2). Quantitative and qualitative methods were used to synthesize the results.

**Results:**

The search identified a total of 1161 articles. Thirty studies from 14 LMICs met the eligibility criteria. On clinical outcomes, 12 and 9 studies reported on glycated hemoglobin (HbA1c )and fasting blood glucose (FBG) respectively. Text messages was the most commonly applied mHealth approach, used in 19 out of the 30 studies. Ten out of the 12 studies (83.3%) that reported on HbA1c had a percentage difference of <0.3% between the mHealth intervention and the comparison group. Additionally, studies with longer intervention periods had higher effect size and percentage difference on HbA1c (1.52 to 2.92%). Patient-centred implementation outcomes were reported variedly, where feasibility was reported in all studies. Acceptability was reported in nine studies, appropriateness in six studies and cost in four studies. mHealth evidence reporting and assessment (mERA) guidelines were not applied in all the studies in this review.

**Conclusion:**

mHealth interventions in LMICs are associated with clinically significant effectiveness on HbA1 but have low effectiveness on FBG. The application of mERA guidelines may standardize reporting of patient-centered implementation outcomes in LMICs.

**Trial registration:**

PROSPERO: Registration ID 154209.

**Supplementary Information:**

The online version contains supplementary material available at 10.1186/s12966-021-01238-0.

## Introduction

Type 2 Diabetes is now a leading public health problem in Low-and Middle Income Countries (LMICs) [1] affecting all age categories and resulting in huge economic implications to healthcare [2, 3]. LMICs are home to 80 % of all people with type 2 diabetes (336 million) [4] and more than 80% of all undiagnosed people with diabetes [2]. It is projected that between 2019 and 2030, the prevalence of type 2 diabetes is likely to increase from 13.5% to 15.0% in LMICs compared to 10.4% to 11.4% in high-income countries [2]. Further, out of the total number of deaths related to diabetes globally, 41.8% and 58.2% occur in Low- and Middle- Income Countries, respectively [2]. The rising prevalence of type 2 diabetes in LMICs is attributed to the nutrition transition, and the increasing prevalence of overweight and obesity. The other factors include, urbanization, cultural and social changes, sedentary lifestyles, changes in diagnostic criteria and screening practices [5–7].

Optimal diabetes management requires a systematic approach, and the involvement of a coordinated, multidisciplinary team that is committed to patient-centered outcomes [8]. It is recommended that clinicians apply a patient-centered approach and minimum clinically important difference (MCID) treatment models by considering the statistical significance and clinical significance of research findings [9]. Essential guidelines for the patient-centered approach include individualized therapy and shared decision-making [10]. Additionally, effective patient-centered diabetes self-management requires the support and promotion of essential self-care behaviors [11]. These behaviors include healthy eating, physical activity, medication usage, monitoring and usage of patient-generated data, prevention, detection and treatment of acute and chronic complications, healthy coping with psychosocial issues and problem solving [12]. These behaviors have been described as Diabetes Self-Management Education and Support (DSMES) domains. Self-management education is linked to clinically important benefits on glycated hemoglobin (HbA1c), and cost of treatment [13–18]. This notwithstanding, self-care in most LMICs is not optimally attained due to disadvantaged access to healthacre and low-quality healthcare, poverty, low literacy levels and incorrect perceptions about diabetes [19–21].

The remarkable increase in ownership and use of mobile phones in LMICs provides a potential opportunity for the application of mobile health (mHealth) in self-care and behavior change interventions for type 2 diabetes [22–24]. mHealth is the medical and public health practice supported by mobile devices, such as mobile phones, patient monitoring devices, personal digital assistants (PDAs), and other wireless devices [25]. Evidence shows that mHealth has the potential to facilitate accessibility and coverage of healthcare services as well as positively influencing clinical outcomes, compliance, self-care practices and quality of life for people with type 2 diabetes [26–29]. Whereas there is close similarity between mHealth and e-health, the later refers to an emerging field that links medical informatics, public health and business, that delivers or enhances health services and information via web-based technologies [30]. However, eHealth heavily relies on internet technology, which limits its applicability in LMICs, due to unreliable access to internet [31].

A recent metanalysis on mHealth interventions for diabetes in LMICs revealed promising but limited evidence on the effectiveness of mHealth interventions on glycemic control [32]. Further, a pooled effect on HbA1c from three studies on mobile phone–based interventions showed a larger effect of 25.46 mmol/mol or 20.50%; (95% CI 20.7 to 20.3%; I2 = 0%) [33]. mHealth interventions have also been found to be cost-effective [34] despite being criticized for having meager user satisfaction ratings coupled with usability challenges [35]. In LMICs, a few studies on mHealth have shown changes in clinical outcomes, adherence and improved communication with providers, decreased travel time, ease to receive expert advice and cost-effective education [36].

Further, evidence from LMICs reveal unique patient circumstances that hinder optimal utilization of mHealth approaches. Inadequate resources, low digital literacy and low health literacy and limited inclusion of motivation techniques hinder optimal utilization of mHealth in LMICs [37]. As such, distinguishing treatment effectiveness or clinical outcomes from implementation effectiveness is important for transferring interventions from experimental settings to the community [38, 39]. This distinction, to the best of our knowledge, has not been examined on mHealth interventions for type 2 diabetes in LMICs.

The objective of this systematic review therefore was to examine the clinical outcomes and patient-centered implementation outcomes of mHealth interventions with a focus on type 2 diabetes in LMICs.

## Methods

### Data sources and registration

This review applied the Preferred Reporting Items for Systematic review and Meta-Analysis (PRISMA) guidelines [40] with the PICOS framing. The review has been registered and amended in PROSPERO https://www.crd.york.ac.uk/prospero/#recordDetails (Registration ID 154209) and funded by VLIR-UOS

(Grant-number: KE2017IUC037A101)

### Search strategy

The search strategy was applied on Cochrane and Web of Science Cochrane Library, Web of Science, PubMed, Scopus, and Ovid databases. (Supplementary File 1). These databases were systematically searched with Boolean combinations of key words and MeSH headings. An electronic search was conducted using the following terms and Boolean Operators: ((mobile health OR mHealth) AND (type 2 diabetes) AND/OR (DSMES) AND/OR (acceptability) AND/OR (appropriateness) AND/OR (feasibility) AND/OR (cost) AND/OR (sustainability)). Acceptability, appropriateness, feasibility, cost and sustainability were based on the definitions in the conceptual framework for implementation outcomes by Proctor *et al.* [38]. We searched for articles published in English between January 2010 and August 2021. Additional records were searched through citations from relevant reviews given that online data bases can be incomplete [41].

### Study selection

This review included randomized controlled trials (RCTs), cluster randomized controlled trials, feasibility studies and prospective observational cohort studies from LMICs. The search also included cohort and follow-up studies of intervention studies that have been published in peer-reviewed journals. Our review was limited to studies that are designed for adults diagnosed with type 2 diabetes. We included studies in which the mHealth intervention was designed to be an enabler for delivery of DSMES for patients with type 2 diabetes [1]. mHealth or mobile health are medical and public health practice supported by mobile devices, such as mobile phones, patient monitoring devices, personal digital assistants (PDAs), and other wireless devices as defined by WHO [25]. DSMES domains include diabetes pathophysiology and treatment options; healthy eating; physical activity; medication usage; monitoring and usage of patient generated health data; prevention, detection, and treatment of acute and chronic complications; healthy coping with psychosocial issues; and problem solving [42]. The selection of studies was conducted by MM and independently reviewed by FK, CM and RV. We excluded studies on children and adolescents, pregnant women, or any other forms of diabetes besides type 2 diabetes such as pre-diabetes, type 1 diabetes or gestational diabetes [43]. We also excluded studies where the mHealth intervention was designed for to support healthcare workers and those studies that did not target the patient.

### Data collection process

Data from all eligible articles was summarized by the first author (MM) and reviewed by the second and third authors (FK & CM) using structured evidence tables (Table 1 & 2). A standardized criterion for data collection was designed by the authors to extract and tabulate relevant study characteristics. These characteristics include study location, study type, duration of study, clinical outcomes (HbA1c and FBG), mHealth intervention and function, DSMES domains and patient-centered implementation outcomes.

**Table 1:** Summary of general study Characteristics

Study and location	Study Design	Sample Size	Participant Age (Years)	Duration of Intervention	mHealth intervention delivery
Anzaldo *et al.* [44] Mexico	RCT	n=301 I1: (Project Dulce)102; I2: (Project Dulce with Technology enhancement) 99 C: 100	18-75	10 months	Arm 1: PD TE: Interactive surveys, text messages, short educational videos Arm 2: PD: combination of care management by a multidisciplinary team led by trained clinicians and nurses, as well as a peer-led group education Bidirectional
Chai *et al*. [45] China	Cohort	n=209	Mean age: 51.97 ± 12.76	4 Months	Upload insulin dosages three or more times for the FPG and postprandial plasma glucose (PPG) on a panel computer
Chao *et al.* [46] China	RCT	n=121; I: 62; C: 59	C: Not provided I: 63.71 (37 -88)	18 months	mobile app; Cloud-based IPMF Bidirectional
Dong *et al* [47] China	RCT	n=120 I: 60; C: 60	18-60 (23-60)	12 months	Web-based app: WeChat platform Bidirectional
Doocy *et al*. [48] Lebanon	Longitudinal Cohort	10 HC; 1020	< 40 ^a^	20 months	mHealth app (PCHR),
Fottrell *et al*. [49] India	CRCT	n=13,728 I1: 4,093; I2: 4,079 C: 5,008;	Mean Age not provided Adults ≥30	18 months,	1. Voice messages 2-weekly (14 months 2. Monthly PLA group meetings (with a 4-phase PLA cycle)
Gunawardena *et al.* [50] Sri Lanka	RCT	n=67 I: 35 C: 32	I: 53 (SD 11), C: 52 (SD 12)	6 months	Android based Smart Glucose Manager (SGM) every 3 months
Goodarzi *et al.* [51] Iran	RCT	n=100 I: 50, C: 50	I:50.98 (SD = 10.32) C: 56.71 (SD = 9.77)	3 months	Text Messages: 4weekly messages Unidirectional
Haddad *et al.* [52] Iraq	Feasibility RCT	n=50	Mean: 51.4 (SD 10.3)	6 months	Text Messages 1 message per week 5 Bidirectional
Huo *et al* [53] China	RCT	n=502 I: 251 C:251	Mean: 59.5 (SD 9.4) I: 59.5 (SD 9.1) C: 59.5 (SD 9.3)	6 months	Text Messages 6 SMSs per week for 6 months; Weekly unidirectional messages Bidirectional
Islam *et al.* [54] Bangladesh	RCT	n=236 I:108; C:108	mean age: SD: 48.1 ± 6 9.7	6 Months	Text messages and Voice calls to the study team for any queries in response to the text message (with a 2-day response). Bidirectional
Kumar *et al.* [55] India	RCT	n=955 I: 479, C: 476	I: 57.5 (SD 10.8) C: 57.0 (SD 10.7)	12 months	Text Messages Patient specific frequency (average of 2 times per month for 12 months) Unidirectional
Li *et al.* [56] China	RCT	n=101 I: 55 C: 46	48.2 (SD 10.4)	3 months	R Plus Health app (Recovery Plus Inc), which connected wirelessly to a chest-worn heart rate band (Recovery Plus Inc) to
Liao *et al* [57]. China	CRCT	n=149 I:69, C: 80 13dancing groups	Mean age: 62	3 months	Wrist-worn activity trackers (Lifesense MAMBO2 wristbands) Activity uploaded to the cloud via a paired smartphone device) Physical activity reports on the paired smartphone via WeChat without support and peers
Limaye *et al.* [58] India	RCT	n=265; I: 132, C: 133	Mean: 36.2 (SD 9.3) I: 36.8 (7.2) C: 35.7 (8.1)	12 months	Text messages: 3 per wk Emails: 2 e-mails/ wk between 1000–1300 h; Website and Facebook Bidirectional 10% of messages required reply
Olmen *et al.* [59] Congo Cambodia Philippines	RCT	N=1471 Congo :506; Cambodia:484; Philippines:481;	Overall: NP I: 58 (SD 10) C: 60 (SD 10)	24 months	Text Messages Congo 5 times/wk; Cambodia: 6 times/wk Philippines: 2 times/week Voice messages Cambodia ¼ of all messages to specific groups, Unidirectional
Owolabi *et al*. [60] South Africa	RCT	n=216 I: 108, C: 108	Overall: 60.64 (SD 11.58)	6 Months	Standard of care plus Tailored Short message services (SMS) Unidirectional
Owolabi *et al*. [61] South Africa	RCT	n=216 I: 108; C: 108	Overall: 60.64 (SD 11.58)	6 Months	Text Messages Daily SMS: 2 times a week; Unidirectional
Patnaik *et al.* [62] India	RCT	n=66 I: 33 C: 33	42.29(SD 9.5)	24 Months	Mobile-based android application Bi-directional
Peimani *et al.* [63] Iran	RCT	n=150 I1: 50 I2: 50 I3: 50	I1:49.78(SD 9.76) I2:53.26 (SD 10.49) C: 54.56 (9.88)	3 months	Text Messages Voice Calls Arm 1: individually tailored SMS: each person received 75% of their messages tailored to 2 reported barriers to adherence Arm 2: non- tailored SMS: random messages sent irrespective of barriers with Voice Calls: Weekly
Pichayapinyo *et al.* [64] Thailand	Cohort	n=35	54.9 (SD:6.3)	4 Months	Interactive Voice Response (IVR) & email content was translated into Thai IVR calls lasting 5–10 minutes each for 12 weeks
Pfammatter *et al.* [65] India	Parallel Cohort	n= 1925 I: 982, C: 943	Overall: 32.2 (SD 10.6) I: 32.83 (SD 9.39) C: 31.66 (SD 11.64)	6 months (Dec 2012-June 2013)	Text Messages (Multilanguage texts) Daily text messages for the first 6 days followed by 2 SMSs per week Unidirectional
Rasoul *et al.* [66] Iran	RCT	n=98 I: 49 C: 49	32.1 (SD 4.9)	5 months	Weblogs Text, video, recorded voice, and nutrition pyramid for diabetic patients 3 days/week, 1:30 hr /session Total: 60 sessions
Rotheram-Borus *et al.* [67] South Africa	Cohort	n=22 (Women)	53 (SD 12.8)	3 months followed by a post-trial FU at 6 months	Text Messaging Diabetes Buddies program: 12 psycho-educational group sessions Daily mobile phone probes on health 3 text messaging to a buddy: Frequency: Daily Bidirectional
Shahid *et al.* [68] Pakistan	RCT	n=440 I: 220 C: 220	I: 48.93 SD(8.83), C 49.21 (SD 7.92)	6 months	Voice calls Calls every 15 days for a period of 4 months: Total of 8 calls
Steinman *et al* [69] Cambodia	CRCT	n=3948 C: 1,737, I: 1,099	NR	12 months	Tablet and mobile voice messages delivered via patient’s mobile phones
Sun *et al.* [70] China	RCT	n=91 I: 44, C: 47	Overall: NP I: 68.04 (66-72)^b^ C: 67.9 (66-71) ^b^	6 months	Mobile Application Bidirectional
Wang *et al*. [71] China	RCT	n=120 I: 60, C:60	Mean age: 45.4	6 months	Mobile application
Yasmin *et al*. [72] Bangladesh	RCT	n=320 I: 160 C: 160	I: 53 [30–85] C: 51 [30–75]	12 months	Personalized Voice calls every 10 days, except Fridays and other national holidays
Zhou *et al.* [73] China	RCT	n=100 I: 50 C: 50	Overall: NP I: 53.5 (SD 12.4) C: 55.0 (SD 13.1)	3 months	Mobile Application Welltang mobile App App for both pts and clinician: 1. Transfers data to servers. Once per week/ Every 2 wks. Feedback: 3-10mins

*App* Application; Personally controlled health record; *PD* Project Dulce–only; *PD-TE* Project Dulce technology enhanced with mobile tools; *PPG* postprandial plasma glucose; *PT* Patient; *QA* Question and answer; *SBP* systolic Blood Glucose; *T2DM* type 2 Diabetes Mellitus ^a^ Mean age not provided; ^b^ Interquartile rage

**Table 2:** Clinical and patient-centred Implementation outcomes

Study	Clinical Outcomes	Patient-centred Implementation outcomes
Anzaldo *et al.* [44]	HbA1c, TC, LDL-c, HDL-c, BMI, SBP, DBP	Feasibility, Appropriateness, Acceptability
Chai *et al*. [45]	FPG ≤ 7 mmol/l, PPG ≤ 10 mmol/l, HbA1c level ≤ 7%.	Feasibility, Appropriateness
Chao *et al.* [46]	Hb, HbA1c, weight, BMI	Feasibility, Appropriateness
Dong *et al.* [47]	FPG, 2hPG, HbA1c	Feasibility
Doocy et al. [48]	HbA1c, BP, FBG	Feasibility
Fottrell *et al.* [49]	PA, BP, HR, Waist Circumference, weight, Height, QoL, Urine Cotinine	Feasibility, Acceptability, Cost
Goodarzi *et al.* [51]	BMI, L-FBG, HbA1c, TC, TG, HDL-C, LDL-C, BUN, Cra, SE, SBP, DBP,	Feasibility, Appropriateness
Gunawardena *et al.* [50]	HbA1c	Feasibility
Haddad *et al.* [52]	Knowledge, HbA1c, cost	Feasibility, Acceptability, Cost, Appropriateness
Huo *et al.* [53]	Primary: HbA1c, Secondary: FBG, LDL, LDL-C, SBP, BMI, PA	Feasibility, Appropriateness, Cost
Islam *et al.* [74]	HbA1c	Feasibility
Kumar *et al.* [55]	FBG, TC, BMI, BP	Feasibility
Li *et al.* [56]	BMI, hemoglobin HbA1c, HOMA-IR, Resting heart rate (bpmc), Step test (bpm) Muscle strength	Feasibility
Liao *et al*. [57]	Heart rate	Acceptability, Feasibility, Appropriateness
Limaye *et al.* [58]	BMI, weight, waist circumference, BP, FBG, LDL-C, HDL-C	Acceptability, Feasibility, Cost, Sustainability
Owolabi *et al.* [60]	Diet adherence, PA adherence	Acceptability, Feasibility, Appropriateness
Owolabi *et al.* [61]	RBS, BMI, SBP, DBP	Acceptability, Feasibility
Patnaik *et al.* [62]	HbA1c	Feasibility, Appropriateness
Peimani *et al.* [63]	HbA1c, FBG, LDL-C, HDL-C, SCI, BMI, DMSES	Feasibility
Pichayapinyo *et al.* [64]	HbA1c, FBG	Feasibility, Acceptability
Pfammatter *et al.* [65]	Fruit, vegetable and fat consumption; Exercises	Feasibility, Acceptability
Rasoul e*t al* [66]	FBG, BMI, SBP, DBP	Feasibility
Rotheram-Borus et al [67]	HbA1c, BMI, BP	Feasibility
Shahid *et al.* [68]	HbA1c, LDL	Feasibility
Steinman *et al.* [69]	FBG, SBP, DBP	Feasibility, Sustainability
Sun *et al* [70]	HbA1c, PBG, FBG, BMI, TG, HDL-C, LDL-C Cr, AST	Feasibility
Wang *et al*. [71]	HbA1c, FPG	Appropriateness, Feasibility
Yasmin *et al*. [72]	FBS: < 7.0 mmol/L, and the PPG 2 h after breakfast < 11.1 mmol/L	Feasibility
Zhou *et al.* [73]	HbA1c, BP, LDL-C, weight, BG, Satisfaction, T2DM knowledge	Feasibility

*AST *aspartate transaminase; *BG* Blood Glucose; *BMI* Body Mass Index; *BP* Blood pressure; *Cr* Creatinine; *DSME* Diabetes Self-Management and Education; *FBG* Fasting Blood glucose; *FBS* Fasting Blood Glucose; *FU* follow-up; *HbA1c* Glycated Haemoglobin; *HDL-c* High Density Lipoprotein Cholesterol; *I1* First Intervention Arm; *I2* Second Intervention Arm; *IPMF* interactive personalized management framework; *IVR* Interactive Voice Response; *LDL-c* Low Density Lipoprotein Cholesterol; *Med* medication; *Mos* Months; *NO* Number of; *NP* Not Provided; *OP* outpatient; *PA* Physical Activity; *PBG* Post prandial Blood Glucose; *PCHR* Personally controlled health record; *TC* Total cholesterol; *TG* Triglycerides

### Quality of studies and risk of bias assessment

To assess quality of the articles, we applied the 2010 CONSORT (Consolidated Standards of Reporting Trials) guidelines [75] and the STROBE (Strengthening the Reporting of Observational Studies in Epidemiology) guidelines [76]. This approach has been used elsewhere to assess the quality of studies [77–79]. An analysis of the quality of the studies included in this review is presented as heat maps (Supplementary File 2). A percentage quality score of >66.6% is rated as high, 50-66.6% as fair and <50% as low. Assessment of quality was conducted by two independent researchers, MM and ES. Additionally, the risk of bias in the included studies was assessed using the Cochrane risk of bias tool for randomized trials (RoB 2) (Supplementary File 3).

### Summary measures

The primary outcome measures in this study are clinical outcomes and patient-centered implementation outcomes for type 2 diabetes mHealth interventions. Specifically, clinical outcomes were synthesized using quantitative methods based on effect sizes of HbA1c and FBG. HbA1c and FBG measure the effectiveness of interventions for the management of type 2 diabetes [80]. Additionally, the percentage difference between the mHealth intervention and the comparison group for HbA1c was analyzed. The patient centered implementation outcomes included acceptability, feasibility, appropriateness, cost and sustainability [38, 81–84]. Acceptability is the perception amongst implementation stakeholders that a particular treatment, service is agreeable, palatable, or satisfactory [33]. Appropriateness is the perceived fit, relevance or compatibility of the innovation for a given practice setting, provider or consumer; and/or perceived fit of the innovation to address a particular issue [33]. Cost is defined as the incremental, implementation or overall costs of delivery based on the settings [33]. Feasibility is the extent to which a new treatment, or an innovation, can be successfully applied or implemented in a specific setting [34]. Sustainability is the extent to which an implemented treatment is maintained within a service setting’s usual or stable operations, as defined by various authors [35–37].

### Synthesis of results

To conduct a quantitative synthesis for clinical outcomes, standardized effect sizes were calculated using two-stage process [85]. In the first stage the effect size of HbA1c and FBG was calculated separately for each study from means and standard deviations. In the second stage, the combined effect size as a weighted average of the intervention effects was derived from the individual studies. The effect sizes were calculated using the formula d = (<post>-<pre>)/stdev to account for between group and within group comparisons. Cohen's d was calculated to derive standardized effect sizes and then converted into Hedges' g to correct for their upwards bias [86]. The magnitude of Hedges' g was interpreted using Cohen's convention where an effect size of < 0.20 is considered to be small, 0.50 to 0.80 as medium, while scores > 0.80 as large [87]. The five patient-centered implementation outcomes were analyzed in an excel spreadsheet and presented descriptively.

## Results

**Fig. 1:**
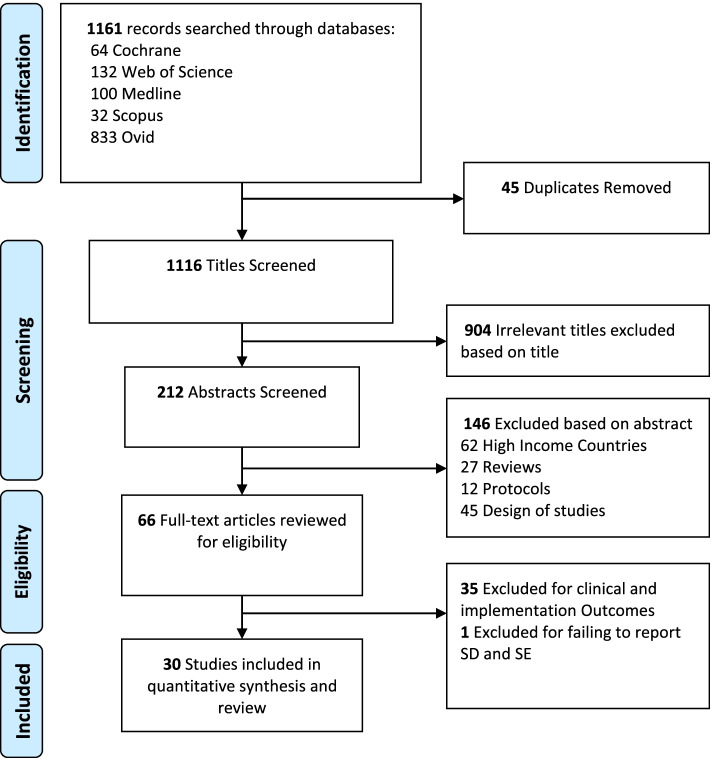
Study selection flow diagram

The search identified a total of 1,161 articles. After removal of duplicates, 1,116 titles of articles were screened and a total of 30 studies that met the eligibility criteria were included in this review (Fig. 1). The 30 eligible studies include 21 randomized controlled trials [44, 46, 47, 50, 51, 53–56, 58–62, 66, 68, 70–74] two feasibility randomized controlled trials [52, 63], three cluster randomized control trials [49, 57, 69] and five cohort studies [45, 48, 64, 65, 67]. The studies were conducted in 14 LMICs including nine in China [45–47, 53, 56, 57, 70, 73, 88], five in India [49, 58, 62, 65, 89], three in South Africa [60, 61, 67], three in Iran [51, 63, 66], two in Bangladesh [49, 54] and one each in Iraq [52], Lebanon [48], Pakistan [68], Mexico [44], Cambodia [69] and Thailand [64]. One other study was multicentre, conducted in Congo, Cambodia and Philippines [59].

### Study quality

The overall mean rating based on these checklists for the randomized controlled trials and cohort studies was 81.8% and 87.7% respectively and categorized as high quality (Supplementary Files 2).

### Study and sample characteristics

The 30 studies included a total of 27,142 participants (Mean =904.7 SD=2548.6) published between 2010-2021, with 66.7% published between 2017 to 2021. The mean duration of the studies was 8.9 months (SD=6.4 min-max: 3-24 months).

### mHealth interventions

Table 3 below categorizes mHealth interventions based on the WHO categorization [25]. In summary text mobile phone text messages (MPTMs) was the most applied mHealth approach, applied in 19 studies. Mobile apps were applied in 10 studies while four studies [50, 56, 57, 70] applied wearable or portable monitoring devices to monitor blood glucose, physical activity or heartbeat rate.

**Table 3:** Categories and functions of mHealth

Category of mHealth	Function	Studies
Mobile technology and devices, including mobile phone text messages (MPTMs)	Knowledge and tips	Dong *et al*. [47], Goodarzi *et al.* [51] Huo *et al.* [53] Islam *et al.* [90]
	Suggestions	Haddad *et al.* [52]; Limaye *et al*. [58] Owolabi *et al.* [61]; Owolabi *et al.* [60]; Peimani *et al.* [63]; Pfammatter *et al*. [65]; Rotheram-Borus *et al.* [67]; Islam *et al.* [90]; Kumar *et al.* [55]
	Reminder	Huo *et al*. [53]
	Medical consultations	None
	Feedback	Huo *et al.* [53]; Haddad *et al.* [52]; Limaye *et al*. [58]; Peimani *et al.* [63]; Rotheram-Borus *et al.* [67]; Islam *et al.* [90]; Liao *et al.* [57]
Telemedicine	Knowledge and tips	Rasoul *et al.* [66]; Chai *et al.* [45]
	Suggestions	Limaye *et al*. [58] ; Rasoul *et al.* [66]
	Reminder	None
	Medical consultations	None
	Feedback	Liao *et al.* [57]
Mobile Phone Calls (MPCs)	Knowledge and tips	Fottrell *et al.* [49]
	Suggestions	Yasmin *et al.* [72]; Shahid *et al*. [68]; Pichayapinyo *et al.* [64]; Steinman *et al.* [69]
	Reminder	Yasmin *et al.* [72]; Shahid *et al*. [68]
	Medical consultations	None
	Feedback	Yasmin *et al.* [72]; Anzaldo-Campos *et al.* [64]
mHealth Apps	Knowledge and tips	Wang *et al* [71]; Li *et al.* [56]
	Suggestions	Chai *et al.* [45]; Sun *et al* [70]; Wang *et al.* [71]; Chao *et al.* [46]
	Medical consultations	Zhou *et al.* [73]; Anzaldo-Campos *et al.* [44]; Patnaik *et al.* [62]
	Reminder	Zhou *et al.* [73]; Gunawardena *et al.* [50]; Liao *et al.* [57]; Li *et al*. [56]; Wang *et al.* [71]
	Data monitoring/ collection/ store/ transmit	Doocy *et al.* [48] ; Patnaik *et al*. [62],
	Feedback	Zhou *et al.* [73]; Anzaldo-Campos *et al.* [44]
Wearable or Portable Monitoring Devices (WPMDs)	Data monitoring/ collection/ store/ transmit	Sun *et al.* [70]; Gunawardena *et al.* [50]; Liao *et al.* [57]; Li *et al*. [56]

### Clinical outcomes of mHealth intervention

To examine clinical outcomes in this review, we examined changes in HbA1c and FBG. HbA1c (mean SD) was reported in 12 studies [44, 46, 47, 51, 53, 56, 63, 64, 68, 70, 71, 73] (Table 4). As summarized in Table 4, one study [47] had a large effect size (Cohen’s d =1.15) while three studies [44, 46, 73] reported a medium effect size (Cohen’s d =0.57). Five of the 12 studies that reported HbA1c had a small effect size (Table 4). Ten out of the twelve studies (83.3%) that reported on HbA1c had a percentage difference of <0.3% between the mHealth intervention and the comparison group. Pichayapinto et al [64] only reported the effect size (Cohen’s d= -0.5) and hence the percentage difference was not calculated. This review found a correlation between studies that used mobile applications approach with medium effect sizes, including Zhou et al. [73] (ES=0.57) Chao et al. [82], (ES=0.58), Anzaldo et al. [44] (ES=0.64). Three studies that used text messages had lower effect sizes, including Peimani *et al.* [63] (ES=0.28) Huo *et al.* [53] (ES=0.36) and Goodarzi *et al.* [51] (ES=0.40). The highest effect size (ES=1.16) in this review was reported by Dong *et al.* [47], a study that used a text messaging platform (WeChat). Studies that used wearable devices had mixed effect sizes, with Li *et al*. [56] reporting the least effect size (ES=0.12), while Sun *et al*. [70] had a medium effect size (ES=0.46).

**Table 4:** Summary of Study Effects Size for HbA1c (%)

Study	mHealth Mode of delivery	Control							Intervention							
		Study Duration (Months)		Pre-intervention		Post-intervention				Pre-intervention				Post-intervention	Effect Size	
			n	Mean	SD	Mean	SD	p-value	n	Mean	SD	Mean	SD	% Pre-Post Intervention Difference	Cohen’s d	Hedge’s g
Anzaldo *et al.* [49]^1^	Mobile App	10	100	10.90	2.02	10.60	3.29	0.01	201	11.29	2.28	8.46	3.31	2.83	0.64	0.64
Chao *et al.* [46]	Mobile App	18	48	8.95	2.34	7.82	1.87	0.03	49	8.44	2.28	6.92	1.27	1.52	0.58	0.58
Dong *et al.* [47]	MPTMs, Int	12	59	9.23	2.13	8.35	1.75	0.05	60	9.55	2.38	6.63	1.17	2.92	1.16	1.16
Goodarzi *et al.* [51]	MPTMs, Uni	3	38	7.91	1.24	7.02	1.02	0.24	43	7.83	1.12	7.48	1.26	0.36	0.40	0.40
Huo *et al*. [53]	MPTMs Int	6	251	6.90	1.40	6.70	1.30	0.00	251	7.10	1.40	7.20	1.50	0.10	0.36	0.36
Li *at al* [56].	Mobile App	3	41	7.50	1.80	6.80	1.33	0.43	44	7.20	1.8	6.65	1.08	0.55	0.12	0.12
Peimani *et al.* [63]^1^	MPTMs, Uni	3	50	7.41	1.40	7.16	1.31	0.19	50	7.52	1.49	7.55	1.44	0.90	0.28	0.28
Pichayapinyo *et al.* [64]	MPCs	3	35	NR	NR	NR	NR	NR	NR	NR	NR	NR	NR	NR	0.50^2^	NR
Shahid *et al.* [68]	MPCs	6	220	9.85	1.37	9.36	1.15	0.001	220	10.09	1.71	8.63	1.29	1.46	0.15	0.15
Sun *et al.* [70]	Mobile APPs WPMDs	6	44	7.84	0.73	6.84	0.76	0.46	47	7.88	0.64	7.22	0.87	0.66	0.47	0.46
Wang *et al*. [71]	Mobile App	6	60	8.68	2.26	7.92	2.15	0.886	60	8.62	2.33	7.12	2.01	1.50	0.38	0.38
Zhou *et al.* [73]	Mobile App	3	50	9.86	2.38	7.91	1.58	0.01	50	9.76	2.51	8.97	2.08	0.79	0.57	0.57

^1^Combined means and *SD *Study had two intervention arms; ^2^ Reported effect size, interquartile range IQR; ^3^NR: Not reported; WPMDs: Wearable or portable monitoring devices; MCPs: Mobile phone calls (MPCs), MPTMs : mobile phone text messages; Uni: Unidirectional; Int: Interactive

Additionally, studies that had longer durations of the intervention [44, 46, 47] had higher effect size and percentage difference (2.83, 1.52 and 2.92) between the intervention group and the comparison group.

Table 5 shows FBG as reported in 9 studies [47, 51, 53, 55, 63, 66, 70, 71, 73]. In summary, this review revealed a small effect size of FBG in eight out of the nine studies. The highest effect size for FBG was reported by Zhou *et al.* [73] with a medium effect (Cohen’s d and Hedge’s g= 0.60). Two studies [47, 55] that had longer intervention durations had lower effect size (Cohen’s d 0.08 and 0.01) for FBG.

**Table 5:** Summary of Study Effects Size for Fasting Blood Glucose (mg/dL)

Study		Control						Intervention							
	Study Duration (Months)	mHealth mode of delivery		Pre-intervention		Post-intervention				Pre-intervention		Post-intervention		Effect Size	
			n	Mean	SD	Mean	SD	p-value	n	Mean	SD	Mean	SD	Cohen's d	Hedge’s g
Dong *et al.* [47]	12	MPTMs Int	59	164.7	46.46	134.9	42.01	0.01	60	173.7	89.64	131.9	28.44	0.08	0.08
Goodarzi *et al*. [51]	3	MPTMs, Uni	43	151.47	55.59	142.00	38.00	0.23	38	161.49	54.15	133.56	36.44	0.23	0.23
Huo *et al*. [53]	6	MPTMs, Int	251	153.15	54.05	154.95	59.46	0.01	251	145.95	48.65	135.14	48.65	0.36	0.36
Kumar *et al.* [55]	12	MPTMs, Uni	476	150.50	62.30	149.20	71.40	0.05	479	163.70	66.90	152.80	66.90	0.01	0.01
Peimani *et al*. [63] ^1^	3	MPTMs, Uni	50	166.94	67.52	165.32	57.85	0.04	50	170.99	70.46	150.18	66.08	0.24	0.24
Rasoul *et al*. [66]	5	Telemedicine	49	252.06	39.58	238.24	40.01	0.0001	49	250.26	50.55	131.08	16.04	0.04	0.04
Sun e*t al*. [70]	6	Mobile Apps	47	140.18	33.33	130.45	44.86	0.96	44	144.14	45.77	130.81	39.10	0.01	0.01
Wang *et al.* [71]	6	Mobile APPs	60	165.8	74.9	143.3	65.3	0.796	60	169.4	77.8	118.4	54.4	0.41	0.41
Zhou *et al.* [73]	3	Mobile APPs	50	160.18	52.07	144.50	39.82	0.01	50	158.92	9.73	124.68	25.05	0.60	0.60

^1^Combined means and SD: Study had two intervention arms; WPMDs : Wearable or portable monitoring devices; MCPs: Mobile phone calls (MPCs), MPTMs : mobile phone text message, Uni: Unidirectional; Int: Interactive

### Patient-centred Implementation outcomes

#### Acceptability of mHealth

Acceptability was reported in nine studies [52, 53, 57, 58, 61, 62, 65, 70]. Three studies [52, 53, 61] reported acceptability as the users’ preferred time to receive text messages. Chao *et al*. [46] conducted a pre- and post-interventional assessments and used the interactive personalized management framework mobile application to assess the participants mental readiness to change behaviour. Fottrell *et al.* [49] used small group discussions prior to all interviews involving men and women attendees and with non-attenders to get consensus on desired community changes. Table 6 below describes various aspects of user-satisfaction reported in these studies. Most of the studies that assessed and reported on user-satisfaction provided scanty details on the findings.

**Table 6:** Acceptability of mHealth Interventions

Study	MHealth intervention	Point and method of measurement of satisfaction	Proportion of respondents	General perceived satisfaction rate	Messages/ content was understandable	Willingness to continue using the mHealth intervention
Haddad *et al* [52]	Text messaging	End of intervention: Questionnaire survey	100%	100%	90.5%	100%
Huo *et al.* [53]	Text Messaging	Last follow-up visit acceptability and utility survey	239 (96.8%)	NR	97.1%	93.7%
Li et *al.* [57]	Mobile app Wearable Activity Trackers	End of intervention: 5-point Likert scale Acceptability questionnaire,	100%	Intervention: 45.2% Control: 40.4%		
Limaye *et al*. [58]	Text Messaging, email, Website, Facebook®	End of intervention: Text messages and Facebook® or website	NR	NR	NR	98.0%
Owolabi *et al*. [61]	Text Messaging	Post-intervention: Questionnaire Survey	98 (90.7%) ^a^	98%	NR	95.9%
Patnaik *et al.* [62]	Mobile app	Post-intervention : Mobile Questionnaire Survey		Diet satisfaction ^c^: 3.21 ± 1.02 Treatment satisfaction^d^ : 13.09 ± 1.01		
Pfammater *et al.* [65]	Text Messaging	End of intervention: Telephone survey	Intervention: 611 (62.2%) Control: 632 (67.0%)	NR	NR	NR
Sun *et al*. [70]	Mobile app	End of intervention Likert scale	100%	90% (6.3/7) ^b^	NR	NR
Zhou *et al.* [73]	Mobile app	Pre- and post-intervention App-based question	NR	84%	NR	NR

^a^Assessment of users’ satisfaction only included participants in the intervention arm; ^b^ Rating based on a 7-scale Likert score; ^c^ Total score: 5; ^d^ Total Score: 15

Only two studies in this review [53, 61] assessed the usefulness of the messages with 94.1% and 90.7% of the participants respectively. Two studies [52, 53] showed that 90.5% and 97.1% of the participants respectively, found the content of the intervention to be understandable. In general, only three studies had included measurement of acceptability as a secondary outcome. Five [52, 53, 58, 61, 70] of the nine studies that assessed acceptability measured the willingness to continue using the mHealth intervention after the study. Willingness to continue using mHealth after the intervention was 100% in Haddad *et al.* [52], 93.7% in Huo *et al.* [53], 98.0% in Limaye *et al* [58]*,* 95.9% in Owolabi *et al.* [61]. In Limaye *et al.* [58]*,* 96% of the participants also acknowledged willingness to recommend the intervention to friends.

#### Feasibility of mHealth interventions

Feasibility of mHealth intervention was examined by the application of any DSMES. Table 7 below summarizes the DSMES applied in the studies. In summary, the most applied DSMES was healthy eating, in 26 studies (86.7%) and physical activity, in 24 studies (80.0%). The most applied combination of DSMES was healthy eating, physical activity, and medication usage, applied in 26, 24 and 23 studies respectively.

**Table 7:** DSMES domains applied in mHealth interventions

Study	DSMES Domains							
	1. Diabetes pathophysiology and treatment options	2. Healthy eating	3. Physical activity	4. Medication usage	5. Monitoring and usage of patient generated health data	6. Preventing, detection and treatment of acute and chronic complications	7. Healthy coping with psychosocial issues	8. Problem solving
Anzaldo *et al. * [44]		•	•	•	•		•	
Chai *et al.* [45]	•	•	•	•	•	•		
Chao *et al.* [46]		•	•	•	•	•	•	
Dong *et al.* [47]	•	•		•	•	•		
Doocy *et al.* [48]					•	•	•	
Fottrell *et al.* [49]	•					•		
Gunawardena *et al.* [50]		•	•	•	•			
Goodarzi *et al.* [51]		•	•	•	•			
Haddad *et al.* [52]		•	•	•	•			
Huo *et al.* [53]			•	•	•		•	
Islam *et al.* [54]	•	•	•	•	•	•		
Kumar *et al.* [55]		•			•			
Li *et al.* [56]		•	•	•				
Liao *et al.* [57]			•					
Limaye *et al.* [58]		•	•	•			•	
Olmen *et al.* [59]	•	•	•	•	•	•	•	•
Owolabi *et al.* [60]		•		•			•	
Owolabi *et al.* [61]	•	•	•	•	•	•	•	•
Patnaik *et al.* [62]	•	•	•	•			•	
Peimani *et al.* [63]		•	•	•	•			
Pichayapinyo *et al.* [64]		•	•					
Pfammatter *et al.* [65]	•	•	•	•	•	•	•	
Rasoul *et al.* [66]	•	•	•	•	•		•	
Rotheram-Borus *et al.* [67]		•	•				•	•
Shahid *et al.* [68]		•	•	•		•	•	
Steinman *et al.* [69]		•	•	•	•		•	
Sun *et al.* [70]		•	•					
Wang *et al.* [71]	•	•	•	•	•	•		
Yasmin *et al.* [72]	•	•	•	•	•	•		
Zhou *et al.* [73]		•	•	•	•			

#### Appropriateness of mHealth interventions

Hermes *et al.* [91] describes objective measurement of appropriateness to be the perceived interventional technology fit with the specific context. Seven studies reported appropriateness variedly. As illustrated in Table 8 below.

**Table 8:** Appropriateness of mHealth Interventions

Study	MHealth intervention	Messages/ content was understandable	Measures of appropriateness
Haddad *et al.* [52]	Text messaging	90.5%	• Received messages at appropriate times: 100%
Huo *et al.* [53]	Text Messaging	97.1%	• Text messaging useful: 94.1% • Participants reported reading: 80% >75% of the messages,
Limaye *et al*. [58]	Text Messaging, email, Website, Facebook®	NR	• Recommend approach to family or friends: 96% • Average adherence at 1 year: 74.5% (Mobile messages: 78.0% e-mails: 71.0%). • Average e-mail opening rate at 6 months: 93%
Owolabi *et al*. [61]	Text Messaging	NR	• Satisfied with the timing of the SMS delivery: 98% • Messages were helpful: 100% • Messages did not stress them in any way: 99%
Patnaik *et al.* [62]	Mobile application		• Treatment satisfaction: 12.94 ± 2.9 out of total score of 15 (86.2%)
Sun *et al*. [70]	mHealth management app	NR	• Convenience for telemedical management: 81% • Helpful in self-monitoring of glucose: 93% • Helpful in glucose diabetes knowledge: 98%

#### Cost of mHealth interventions

Four studies [49, 52, 53, 58] analysed the cost of the mHealth intervention (Table 9). Various aspects of cost were reported targeting the patient, the program, or the general population.

**Table 9:** Cost of mHealth interventions

Study	Cost description	Cost	Focus
Huo *et al.* [53]	Cost per text message	US$0.01	Patient
Haddad *et al.* [52]	Cost per text message	€ 0.065	Patient
Fottrell *et al.* [49]	Total annual costs of the PLA intervention	$ 601,484	Program
	Average annual costs of the PLA intervention	$240,594	Program
	Total annual costs of mHealth intervention	$312,630	Program
	Average annual costs of mHealth intervention	$125,052	Program
	Average annual costs of the PLA per beneficiary	$14	Patient/ Program
	Average annual costs of mHealth per beneficiary	$7	Patient/ Program
	Cost-effectiveness ratios for PLA per case of intermediate hyperglycaemia or type 2 diabetes	$316 ($124 per DALY averted)	Population
	Cost-effectiveness ratios per case of type 2 diabetes prevented	$65,18 ($2,551 per DALY averted)	Population
Limaye *et al.* [58]	Annual direct medical cost per participant in the control group	£23.30	Patient
	Annual direct medical cost per participant in the intervention group	£35.80	Patient
	Incremental cost of treating or preventing one case of overweight/obesity in 1 year	£112.30	Patient/ Program

*PLA *Participatory Learning Activities

#### Sustainability of mHealth *intervention*

As shown in Table 10 below, only two studies reported on sustainability of the mHealth interventions [58, 70]

**Table 10:** Sustainability of mHealth Interventions

Study	Indicator	Period of mearing Sustainability	Indicators of sustainability
Limaye *et al.* [58]	Weight, waist circumference, diastolic blood pressure	After 1 year	• Exercise ≥ 150mins/week • Raw food Consumption ≥ 8 servings/week • Energy Dense food consumption ≥ 4 servings/week • Awareness score ≥ 75%
Sun *et al.* [70]	mHealth and eHealth intervention	After the intervention (6 months)	• Cost of implementation and maintenance too high • Low impact shown by the study

## Discussion

This systematic review found clinically significant effectiveness of mHealth interventions on HbA1c in most interventions for type 2 diabetes in LMICs. Ten out of 12 studies had a >0.3% difference for HbA1c between the mHealth intervention group and comparison group. There was however low effectiveness of mHealth on FBG in most interventions, with 8 out of 9 studies that reported FBG showing an effect size of <0.05. Mobile phone text messages (MPTMs) and mobile apps was the most common mHealth approach in 19 and 10 out of 30 studies respectively. Voice calls and wearable devices were used in five and two studies respectively. Despite the popularity of MPTMs in most interventions in our review, this mode of mHealth was associated with lower effectiveness on HbA1c and FBG. Among the patient centered outcomes, feasibility, based on DSMES domains was reported in all studies. There was substantial heterogeneity in reporting of acceptability, appropriateness, cost, and sustainability.

A change of 0.3% (3 mmol/mol) in HbA1c denotes a clinically significant margin and is generally considered to be an acceptable change [92]. Although this change seems to be relatively small, this difference in HbA1c has been associated with clinically significant effects, including reduction in the risk to diabetic complications, lower long-term risk to microvascular complications and all-cause mortality [92–94]. Despite the heterogeneity, our findings indicate that mHeath can be an effective tool to improve HbA1c. On the contrary, studies in this review revealed low effectiveness of mHealth on FBG. These findings concur with a recent metanalysis consisting of nine studies drawn from LMICs and high-income countries that reported a pooled effect size of −0.39; (P<.001) despite the different populations targeted [95]. FBG is known to be affected by numerous factors, that could be attributable to the low effectiveness found in our review [96]. Another interesting finding from this review is that studies with intervention durations of >10 months had a higher percentage change on HbA1c compared to those conducted for shorter periods of time. On the contrary, longer durations were associated with lower effect size for FBG. Longer interventions have been associated with increased engagement, and effectiveness of mHealth interventions [97]. The reasons for the lower effect sizes for FBG are unclear, but could equally be linked with intervening factors that cannot be controlled during the interventions [96].

Patient-centered implementation outcomes included in this review were acceptability, appropriateness, feasibility, cost, and sustainability. Acceptability is associated with user-satisfaction [98]. Studies in our review hardly reported on most patient centered outcomes. Reporting of these outcomes was also widely varied. None of the studies in this review applied the mHealth evidence reporting and assessment (mERA) guidelines [99] in reporting its findings. mERA guidelines provide a criteria to identify minimum sets of information needed to the define the mHealth intervention, where it is implemented, and how it is implemented to facilitate a possible replication of an intervention. mERA guidelines recommend that interventions report on appropriateness of the interventions, user opinions on content or user interface, perceptions about usability, access, cost assessment and connectivity. This review showed that only 4 out of the 30 studies (13.3%) reported on the cost aspects of the intervention. Further, six studies reported on various aspects of appropriateness including assessments on appropriate timing of messages, satisfaction, and convenience of the intervention. In this review, we described feasibility of the intervention based on the DSMES domain applied. Three DSMES domains, including healthy eating, physical activity, and medication usage in this review were associated with a difference of >0.3% for HbA1c . Similar findings have been reported in a review or reviews that linked the application of technology enabled DSME domains to significantly improvement of HbA1c [100]. Additionally, Muller e*t al*. [101]. Found that mHealth interventions can be effective in promoting physical activity and healthy diets in low income settings.

## Strengths and limitations

This review has the strength that we used clearly defined inclusion and exclusion criteria and conducted searches in two phases. However, this review has some limitations. First, most studies in this review did not report on HbA1c, which is considered as the gold standard clinical outcome in diabetes care. Secondly, patient centered implementation outcomes are mainly reported in grey literature, which were not included in our review. Thirdly, most of the studies in this review did not apply the mERA guidelines, hence reducing replication of the intervention. Finally, we only included articles published in the English language, which introduced the language bias.

## Conclusion

mHealth interventions in LMICs are associated with clinically significant effectiveness on HbA1c but have low effectiveness on FBG. Interventions applying mobile apps have a high effect size on HbA1c compared to those that apply text messaging, voice calls or wearable devices. Percentage changes of >0.3% in HbA1c was correlated with three DSMES domains, including healthy eating, physical activity, and medication usage. The use of the mHealth evidence reporting and assessment (mERA) guidelines may standardize and improve reporting of patient-centered implementation outcomes in LMICs.

## Implications for future research

Clinical and patient centered implementation outcomes should be considered in the planning, implementation and monitoring of mHealth interventions. This approach optimizes the individualization of care, which is vital in diabetes care. Additionally, mERA guidelines need to be applied in reporting so as to standardize and provide rigor in mHealth intervention globally.

## Supplementary Information

**Additional file 1.**

**Additional file 2.**

**Additional file 3.**

## Data Availability

All data generated or analyzed during this study are included in this published article [and its supplementary information files].

## Abbreviations

App: Application
AST: aspartate transaminase
BG: Blood Glucose
BMI: Body Mass Index
BP: Blood pressure
CONSORT: Consolidated Standards of Reporting Trials
DSME: Diabetes Self-Management and Education
DSMES: Diabetes self-management education and support
FBG: Fasting blood glucose
FU: follow-up
HbA1c: Glycated haemoglobin
HDL-c: High Density Lipoprotein Cholesterol
I1: First Intervention Arm
I2: Second Intervention Arm
IPMF: Interactive personalized management framework
LDL-c: Low Density Lipoprotein Cholesterol
LMICs: Low- and middle-income countries
Med: Medication
Mos: Months
NO: Number of
NP: Not Provided
OP: Outpatient
PA: Physical Activity
PBG: Post-prandial Blood Glucose
PCHR: Personally Controlled Health Record
PICOS: Participants, interventions, comparisons and outcome(s) and study design
PRISMA: Preferred Reporting Items for Systematic review and Meta-Analysis
PT: Patient
QA: Question and answer
RCTs: Randomized controlled Trials
SBP: Systolic Blood pressure
SD: Standard Deviation
STROBE: Strengthening the Reporting of Observational Studies in Epidemiology
TC: Total cholesterol
TG: Triglycerides
Wk: Week
Wt: Weight
