# Supplementary material for: Clinical and patient-centered implementation outcomes of mHealth interventions for type 2 diabetes in low-and-middle income countries: a systematic review
Source: Int J Behav Nutr Phys Act. 2022 Jan 6;19:1. doi: 10.1186/s12966-021-01238-0 (PMC8734304; doi:10.1186/s12966-021-01238-0)
Supplement: Supplementary file 1 — Additional file 1. [file 12966_2021_1238_MOESM1_ESM.docx]

**Supplementary File 1: Search strategy**

**Cochrane Search**

**ID Search Hits**

#1 ("type 2 diabetes"):ti,ab,kw with Publication Year from 2010 to 2021, in Trials 27729

#2 mobile health 6976

#3 mhealth with Publication Year from 2010 to 2021, in Trials 1731

#4 healthy eating with Publication Year from 2010 to 2021, in Trials 4096

#5 physical activity with Publication Year from 2010 to 2021, in Trials 38877

#6 medication usage with Publication Year from 2010 to 2021, in Trials 1251

#7 monitoring and usage of patient data 784

#8 prevention, detection and treatment of acute and chronic complication with Publication Year from 2010 to 2021, in Trials 5

#9 healthy coping with psychosocial issues with Publication Year from 2010 to 2021, in Trials 10

#10 problem solving with Publication Year from 2010 to 2021, in Trials 3746

#11 acceptability with Publication Year from 2010 to 2021, in Trials 13313

#12 appropriateness with Publication Year from 2010 to 2021, in Trials 1351

#13 feasibility with Publication Year from 2010 to 2021, in Trials 35035

#14 cost with Publication Year from 2010 to 2021, in Trials 41210

#15 sustainability with Publication Year from 2010 to 2021, in Trials 2144

#16 low income country and middle income country with Publication Year from 2010 to 2021, in Trials 616

#17 middle income country 1686

#18 #1 AND #2 OR #3 with Publication Year from 2010 to 2021, in Trials 1971

#19 #4 OR #5 OR #6 OR #7 OR #8 OR #8 OR #9 OR #10 46743

#20 #11 OR #12 OR #13 OR #14 OR 15 with Publication Year from 2010 to 2021, in Trials 261429

#21 #18 AND #19 OR #20 with Publication Year from 2010 to 2021, with Cochrane Library publication date Between Jan 2010 and Aug 2021, in Trials 258986

#22 #16 AND #21 with Publication Year from 2010 to 2021, in Trials 64

**Search Hits: 64**

**Web of Science**

type 2 diabetes (Title) and mobile Health (All Fields) or mhealth (All Fields) and Diabetes Self management education and support (Abstract) or health eating (Abstract) or physical activity (Abstract) or medication usage (Abstract) or monitoring and usage of patient-generated data (Abstract) or prevention, AND detection AND treatment AND of AND acute AND chronic AND complications (Abstract) or coping AND with AND psychosocial AND issues (Abstract) and Acceptability (Abstract) or Appropriateness (Abstract) and Feasibility (Abstract) and cost (Abstract) and sustainability (All Fields) and Articles (Document Types) and IRELAND or SINGAPORE or PAKISTAN or HUNGARY or ARGENTINA or VIETNAM or BANGLADESH or ETHIOPIA or TUNISIA or GHANA or KENYA or MOROCCO or COSTA RICA or SRI LANKA or IRAQ or UGANDA or ALGERIA or KUWAIT or TANZANIA or CAMEROON or ZIMBABWE or SUDAN or SENEGAL or MOZAMBIQUE or MALAWI or BOTSWANA or GUATEMALA or PALESTINE or COTE IVOIRE or ZAMBIA or ALBANIA or CAMBODIA or LIBYA or SYRIA or RWANDA or YEMEN or NAMIBIA or AFGHANISTAN or LAOS or REP CONGO or ANGOLA or SIERRA LEONE or GUINEA or SAMOA or TOGO or GAMBIA or TONGA or HAITI or YUGOSLAVIA or ERITREA or ESWATINI or GUYANA or BURUNDI or GUINEA BISSAU or SENEGAMBIA or BAHAMAS or CHAD or CENT AFR REPUBL or SWAZILAND or LIBERIA or MALI or NIGER or PAPUA N GUINEA or MYANMAR or BENIN or HONG KONG or PARAGUAY or BURKINA FASO or NEPAL or PERU or ECUADOR or NIGERIA or COLOMBIA or THAILAND or INDONESIA or SAUDI ARABIA or ROMANIA or MEXICO or IRAN or INDIA or BRAZIL or PEOPLES R CHINA (Countries/Regions) and Articles (Document Types) and 2010 or 2011 or 2012 or 2013 or 2014 or 2015 or 2016 or 2017 or 2018 or 2019 or 2020 or 2021 or 2022 (Publication Years) and Articles (Document Types) and English (Languages) and Nutrition Dietetics or Health Care Sciences Services or Behavioral Sciences (Research Areas) and MEETING OF THE NUTRITION SOCIETY IRISH SECTION CONFERENCE ON CHILDHOOD NUTRITION AND OBESITY CURRENT STATUS AND FUTURE CHALLENGES SYMPOSIUM 1 ON CURRENT STATUS or 13TH EUROPEAN NUTRITION CONFERENCE ON MALNUTRITION IN AN OBESE WORLD EUROPEAN PERSPECTIVES OF FEDERATION OF EUROPEAN NUTRITION SOCIETIES FENS SYMPOSIUM 3B ON SARCOPENIC OBESITY METABOLISM AND MECHANISMS or 15TH INTERNATIONAL CONGRESS ON RENAL NUTRITION AND METABOLISM ICRNM or 24TH ANNUAL MEETING OF THE SOCIETY FOR THE STUDY OF INGESTIVE BEHAVIOR SSIB or 25TH ANNUAL MEETING OF THE SOCIETY FOR THE STUDY OF INGESTIVE BEHAVIOR SSIB or 28TH INTERNATIONAL CONGRESS OF PEDIATRICS or 2ND FORUM ON CHILD OBESITY INTERVENTIONS or 4TH ANNUAL WEIGHT AND STIGMA CONFERENCE or 4TH ASIAN NETWORK SYMPOSIUM ON NUTRITION JOINT IUNS WORKSHOP ON CAPACITY AND LEADERSHIP DEVELOPMENT IN NUTRITION SCIENCES IUNS 57TH ANNUAL ASSEMBLY OF THE JAPANESE SOCIETY OF NUTRITION AND DIETETICS or 7TH ANNUAL SCIENTIFIC CONFERENCE ON HYDRATION FOR HEALTH or ANNUAL POSTGRADUATE MEETING OF THE NUTRITION SOCIETY IRISH SECTION or INTERNATIONAL CONFERENCE ON THE POWER OF PROGRAMMING DEVELOPMENTAL ORIGINS OF HEALTH AND DISEASE or JOINT SCIENTIFIC MEETING OF THE AUSTRALIAN AND NEW ZEALAND OBESITY SOCIETY ANZOS AND THE OBESITY SURGERY SOCIETY OF AUSTRALIA AND NEW ZEALAND OSSANZ IN CONJUNCTION WITH THE ASIA OCEANIA ASSOCIATION FOR THE STUDY OF OBESITY AOASO or JOINT WINTER MEETING OF THE NUTRITION SOCIETY AND THE ROYAL SOCIETY OF MEDICINE CONFERENCE ON DIET NUTRITION AND MENTAL HEALTH AND WELLBEING or MEETING OF THE NUTRITION SOCIETY IRISH SECTION CONFERENCE ON CHILDHOOD NUTRITION AND OBESITY CURRENT STATUS AND FUTURE CHALLENGES SYMPOSIUM 4 ON STRATEGIES FOR REDUCING CHILDHOOD OBESITY or NUTRITION SOCIETY SUMMER MEETING CONFERENCE ON CARBOHYDRATES IN HEALTH FRIENDS OR FOES NUTRITION SOCIETY PUBLIC HEALTH NUTRITION MEDAL LECTURE or SYMPOSIUM ON NUTRITION GETTING THE BALANCE RIGHT IN 2010 or SYMPOSIUM ON THE CHALLENGE OF TRANSLATING NUTRITION RESEARCH INTO PUBLIC HEALTH NUTRITION (Exclude – Conference Titles)

**Search Hits: 32 Articles**

**Medline**

(((((((((((type 2 diabetes[Title])) AND (mobile health[Title])) AND (diabetes self-management[Title/Abstract] AND support[Title/Abstract])) OR (health eating[Title/Abstract])) OR (monitoring[Title/Abstract] AND usage of patient-generated data[Title/Abstract])) OR (prevention, detection[Title/Abstract] AND treatment of acute[Title/Abstract] AND chronic complications[Title/Abstract])) OR (healthy coping with psychosocial issues[Title/Abstract])) OR (problem solving[Title/Abstract])) OR (Acceptability[Title/Abstract])) OR (appropriateness[Title/Abstract])) OR (efficacy[Title/Abstract])) OR (cost[Title/Abstract])) OR (sustainability[Title/Abstract])

Filters: Publication Date: 2010-2021; Low-income countries; Clinical Trial, Randomized Controlled Trial

**Search Hits: 100 Articles**

**Scopus Search**

( TITLE ( type 2 diabetes ) AND TITLE-ABS-KEY ( mobile AND health ) OR ALL ( mhealth ) AND ALL ( healthy AND eating ) OR ALL ( physical AND activity ) OR ALL ( medication AND usage ) OR ALL ( monitoring AND usage AND of AND patient-generated AND data ) OR ALL ( prevention, AND detection AND treatment AND of AND acute AND chronic AND complications ) OR ALL ( coping AND with AND psychosocial AND issues ) OR ALL ( problem AND solving ) AND ALL ( acceptability ) OR ALL ( appropriateness ) OR ALL ( feasibility ) OR ALL ( cost ) OR ALL ( sustainability ) ) AND ( EXCLUDE ( PUBYEAR , 2000 ) ) AND ( LIMIT-TO ( AFFILCOUNTRY , "China" ) OR LIMIT-TO ( AFFILCOUNTRY , "Bangladesh" ) OR LIMIT-TO ( AFFILCOUNTRY , "Iran" ) OR LIMIT-TO ( AFFILCOUNTRY , "Singapore" ) OR LIMIT-TO ( AFFILCOUNTRY , "Brazil" ) OR LIMIT-TO ( AFFILCOUNTRY , "Kuwait" ) OR LIMIT-TO ( AFFILCOUNTRY , "Malawi" ) OR LIMIT-TO ( AFFILCOUNTRY , "Nigeria" ) OR LIMIT-TO ( AFFILCOUNTRY , "Mexico" ) OR LIMIT-TO ( AFFILCOUNTRY , "South Africa" ) OR LIMIT-TO ( AFFILCOUNTRY , "Taiwan" ) OR LIMIT-TO ( AFFILCOUNTRY , "Thailand" ) OR LIMIT-TO ( AFFILCOUNTRY , "Botswana" ) OR LIMIT-TO ( AFFILCOUNTRY , "Cameroon" ) OR LIMIT-TO ( AFFILCOUNTRY , "Ghana" ) OR LIMIT-TO ( AFFILCOUNTRY , "Iraq" ) OR LIMIT-TO ( AFFILCOUNTRY , "Lebanon" ) OR LIMIT-TO ( AFFILCOUNTRY , "Namibia" ) OR LIMIT-TO ( AFFILCOUNTRY , "Sri Lanka" ) OR LIMIT-TO ( AFFILCOUNTRY , "Sudan" ) OR LIMIT-TO ( AFFILCOUNTRY , "Zambia" ) OR LIMIT-TO ( AFFILCOUNTRY , "Zimbabwe" ) )

**Search Hits: 32 Articles**

**Ovid Search**

( TITLE ( type 2 diabetes ) AND TITLE-ABS-KEY ( diabetes AND self AND management AND education AND support ) OR TITLE-ABS-KEY ( healthy AND eating ) OR TITLE-ABS-KEY ( physical AND activity ) OR TITLE-ABS-KEY ( medication AND usage ) OR TITLE-ABS-KEY ( monitoring AND of AND patient AND data ) OR TITLE-ABS-KEY ( prevention, AND detection AND treatment AND of AND acute AND chronic AND complications ) OR TITLE-ABS-KEY ( coping AND with AND psychosocial AND issues ) OR TITLE-ABS-KEY ( problem AND solving ) AND TITLE-ABS-KEY ( acceptability ) AND TITLE-ABS-KEY ( appropriateness ) OR TITLE-ABS-KEY ( efficacy ) OR TITLE-ABS-KEY ( cost ) OR TITLE-ABS-KEY ( sustainability ) ) AND ( LIMIT-TO ( AFFILCOUNTRY , "China" ) OR LIMIT-TO ( AFFILCOUNTRY , "Brazil" ) OR LIMIT-TO ( AFFILCOUNTRY , "India" ) OR LIMIT-TO ( AFFILCOUNTRY , "Pakistan" ) OR LIMIT-TO ( AFFILCOUNTRY , "Rwanda" ) OR LIMIT-TO ( AFFILCOUNTRY , "Singapore" ) OR LIMIT-TO ( AFFILCOUNTRY , "Sri Lanka" ) OR LIMIT-TO ( AFFILCOUNTRY , "Thailand" ) ) AND ( LIMIT-TO ( DOCTYPE , "ar" ) )

**Search Hits: 883**
