# Supplementary material for: Clinical and patient-centered implementation outcomes of mHealth interventions for type 2 diabetes in low-and-middle income countries: a systematic review
Source: Int J Behav Nutr Phys Act. 2022 Jan 6;19:1. doi: 10.1186/s12966-021-01238-0 (PMC8734304; doi:10.1186/s12966-021-01238-0)
Supplement: Supplementary file 2 — Additional file 2. [file 12966_2021_1238_MOESM2_ESM.docx]

**Supplementary File 2: Study Quality**

1. CONSORT Study Quality Analysis

1. STROBE Study Quality Analysis
