# Supplementary material for: Clinical and patient-centered implementation outcomes of mHealth interventions for type 2 diabetes in low-and-middle income countries: a systematic review
Source: Int J Behav Nutr Phys Act. 2022 Jan 6;19:1. doi: 10.1186/s12966-021-01238-0 (PMC8734304; doi:10.1186/s12966-021-01238-0)
Supplement: Supplementary file 3 — Additional file 3. [file 12966_2021_1238_MOESM3_ESM.docx]

**Supplementary File 3. Risk of Bias Assessment for Randomized Controlled Trials**

| **Study** | Domain 1a. Randomization Process | Domain 1b. Timing of identification or recruitment of participants | Domain 2. Deviations from intended interventions | Domain 3. Missing Outcome data | Domain 4. Measures of Outcome | Domain 5. Selection of reported result | Overall bias |
| --- | --- | --- | --- | --- | --- | --- | --- |
|  | A | B | C | D | E | F | G |
| Anzaldo *et al.* (66) |  |  |  |  |  |  |  |
| Chao et al (57) |  |  |  |  |  |  |  |
| Dong *et al* (58) |  |  |  |  |  |  |  |
| Fottrell et al. (67) |  |  |  |  |  |  |  |
| Gunawardena *et al.* (72) |  |  |  |  |  |  |  |
| Goodarzi et al. (74) |  |  |  |  |  |  |  |
| Huo *et al* (59) |  |  |  |  |  |  |  |
| Islam *et al.* (90) |  |  |  |  |  |  |  |
| Kumar *et al.* (68) |  |  |  |  |  |  |  |
| Li *et al.*(62) |  |  |  |  |  |  |  |
| Liao *et al* . (60) |  |  |  |  |  |  |  |
| Limaye *et al.* (70) |  |  |  |  |  |  |  |
| Olmen *et al.* (87) |  |  |  |  |  |  |  |
| Owolabi et al.(83) |  |  |  |  |  |  |  |
| Owolabi et al. (82) |  |  |  |  |  |  |  |
| Patnaik *et al.*(69) |  |  |  |  |  |  |  |
| Peimani *et al.* (76) |  |  |  |  |  |  |  |
| Rasoul e*t al* (84) |  |  |  |  |  |  |  |
| Shahid *et al.* (86) |  |  |  |  |  |  |  |
| Steinman *et al* (78) |  |  |  |  |  |  |  |
| Sun *et al* (61) |  |  |  |  |  |  |  |
| Wang *et al*.(88) |  |  |  |  |  |  |  |
| Yasmin *et al*. (89) |  |  |  |  |  |  |  |
| Zhou et al. (63) |  |  |  |  |  |  |  |

1. Domain 1a. Randomization Process
2. Domain 1b. Timing of identification or recruitment of participants
3. Domain 2. Deviations from intended interventions
4. Domain 3. Missing Outcome data
5. Domain 4. Measures of Outcome
6. Domain 5. Selection of reported result
7. Overall bias

**Key**

|  | Low risk of bias |
| --- | --- |
|  | High risk of bias |
|  | Unclear risk of bias |
